# Using the UKROC dataset to make the case for resources to improve cost-efficiency in neurological rehabilitation

**DOI:** 10.3109/09638288.2012.670042

**Published:** 2012-04-16

**Authors:** Lynne Turner-Stokes, Rob Poppleton, Heather Williams, Katie Schoewenaars, Derar Badwan

**Affiliations:** ^1^King’s College London School of Medicine, Department of Palliative Care Policy and Rehabilitation, London, UK; ^2^Regional Rehabilitation Unit, Northwick Park Hospital, Middlesex, UK; ^3^Royal Leamington Spa Hospital for Rehabilitation, Warwickshire, UK

**Keywords:** Cost-efficiency, costs and cost analysis, outcome measures, rehabilitation

## Abstract

**Purpose:**

A key challenge for providers and commissioners of rehabilitation services is to find optimal balance between service costs and outcomes. This article presents a “real-lifeâ application of the UK Rehabilitation Outcomes Collaborative (UKROC) dataset. We undertook a comparative cohort analysis of case-episode data (n = 173) from two specialist neurological rehabilitation units (A and B), to compare the cost-efficiency of two service models.

**Key messages:**

(i) Demographics, casemix and levels of functional dependency on admission and discharge were broadly similar for the two units. (ii) The mean length of stay for Unit A was 1.5 times longer than Unit B, which had 85% higher levels of therapy staffing in relation to occupied bed days so despite higher bed-day costs, Unit B was 20% more cost-efficient overall, for similar gain. (iii) Following analysis, engagement with service commissioners led to successful negotiation of a business plan for service reconfiguration with increased staffing levels for Unit A and further development of local community rehabilitation services.

**Conclusion:**

(i) Lower front-end service costs do not always signify optimal cost-efficiency. (ii) Analysis of routinely collected clinical data can be used to engage commissioners and to make the case for resources to maximise efficiency and improve patient care.

## Introduction

In the current climate of ever-increasing financial pressure, clinicians, purchasers and providers are all under obligation to provide services that both meet the needs of the patients and offer the best value for money. In an earlier article in this issue, we presented the UK Rehabilitation Outcomes Collaborative (UKROC) database and the approach we have taken to engaging the hearts and minds of clinicians in data collection that will support outcome evaluation in relation to patients needs and the inputs provided [1]. However, data collection is only the first step. We also need to engage providers and purchasers to understand the information and to use this to make decisions regarding service provision and allocation of resources, in order to develop effective and cost-efficient rehabilitation services that are sensitive to the needs of their local population.

**Implications for Rehabilitation**

- A key challenge for the provision of rehabilitation services is to strike a cost-effective balance between outcome and service cost, particularly for highly complex cases.
- This article presents a “real-lifeâ application of the UK Rehabilitation Outcomes Collaborative (UKROC) dataset from two tertiary neurological rehabilitation services to demonstrate how the dataset may be used to compare the cost-efficiency of different service models.
- Analysis of routinely collected clinical data can be used to engage commissioners and to make the case for resources to maximize efficiency and improve patient care.

A key challenge for the provision of rehabilitation services is to strike a cost-effective balance between outcome and service cost, particularly for highly complex cases. Early intensive rehabilitation is expensive to provide, but is shown to be cost-effective in reducing length of stay [2]. However, reducing length of stay does not always equate to cost-efficiency. Evidence from the US and other countries has shown that the introduction of fixed episode payment schemes in rehabilitation may lead to poorer functional outcomes [3] and increased rates of discharge to institutional care [4], due to pressure to discharge patients early when funding ceases. Clearly, a balance must be found between rapid throughput and functional gain. For some highly dependent patients with more complex rehabilitation needs, longer lengths of stay are shown to be cost-efficient – the initial investment being offset many times by long-term savings in the cost of ongoing care [5,6]. Payment models for rehabilitation therefore need to be sufficiently flexible to meet the higher costs of rehabilitation for complex cases, provided such payments can be justified by demonstrable cost-efficiency.

The willingness of purchasers to invest in rehabilitation to reduce the cost of long-term care will to some extent depend on who pays for the latter. The UK National Health Service (NHS) provides the most comprehensive state-funded healthcare system in the world, and a closely integrated social services system provides lifelong care and support for individuals who are unable to pay for it themselves. In light of this state responsibility for ongoing care, longer stays in rehabilitation have traditionally been sanctioned for highly complex cases. Until recently, however, there has been no attempt to gather systematic data to evaluate the effectiveness of this approach or to compare different models of care, in order to determine the optimum balance between treatment intensity and length of stay.

The recent introduction of the UK Department of Health’s Payment by Results programme [7] has generated the need for more accurate casemix and costing information. The UKROC database has been set up to provide information on casemix and costing models that will inform the development of national tariffs [8] and also to provide information for benchmarking and outcome evaluation. However, some service providers have felt an initial reluctance to share their data for fear that it may be misinterpreted. In particular, many have expressed concern about the sensitivity of standardised instruments within the dataset, such as the Functional Assessment Measure [9], to reflect the true benefits of rehabilitation – especially in the more dependent patients who tend to fall beneath the floor of this scale.

In this article, we present a “real-lifeâ application of the UKROC dataset from two tertiary neurological rehabilitation services carrying a highly complex caseload to demonstrate how the dataset may be used to compare the cost-efficiency of different service models, and to make the case for resources to maximise efficiency and to improve patient care.

## Methods

### The UKROC dataset

The centralised UKROC database records de-identified data for inpatient episodes from specialist neurological rehabilitation services across the UK. The full 30-item UKROC dataset is described elsewhere [8] and details of its constituent tools are given in a separate article in this issue [1]. Staff are trained in the application of these tools through a national training programme.

### Costing methodology

The UKROC programme also collates data on service costs, according to a standard costing model again described elsewhere [8]. Contributing provider units submit information on their service costs, based on retrospective analysis of their budget statements and accounting costs. Reported costs are then verified (by site visits and follow up correspondence from both expert advisors and analysts) to ensure a consistent approach to cost definition, attribution and allocation. A standard template has been devised for attributing individual lines within the budget statement to different cost types, based on the Department of Health’s Patient Level Costing Standards. Costs are collated under three main cost types – “direct costs,â “indirect costsâ and “overheads.â A standard Service Profile questionnaire is also completed by the provider unit, to supply information regarding the type and nature of service provided, special facilities and staffing levels (discipline and grade).

### Settings

In this study, we present a comparison of data extracted from the UKROC dataset for two “Level 1â specialised neurological rehabilitation services in the UK. According to the UK National Definition Set for Specialised Services [10], a Level 1 service carries a high proportion of complex cases and serves a catchment population of >1 million.

- Unit A is a 30-bed Level 1 unit, serving a catchment population in excess of 5 million in the Midlands area of England.
- Unit B is a 22-bed Level 1 unit, serving a catchment population in excess of 12 million across London and South East England.

Both units provide rehabilitation for a mixed population of neurological disorders, with the emphasis on acquired brain injury. As part of their practice, both also provide a specific service for the evaluation of patients in vegetative and minimally conscious states. They therefore include a proportion of profoundly impaired clients that are not expected to make significant functional gains during the programme. In this sense, the two units were anticipated to carry a comparable caseload.

### Data extraction

Episode data were extracted for all patients admitted to the two services during the 1-year period between 1st April 2008 and 31st March 2009. Service profile and costing information were assembled for the corresponding period.

In this analysis, we present data for levels of input using the Rehabilitation Complexity Score (RCS Version 2) [11]; and for outcome using the Functional Assessment Measure, UK Version (UK FIM+FAM) [9], recorded within the first 10 days of admission to the unit, and within the last 7 days before discharge.

- The RCS is a simple 16-point scale (score range 0–15), comprising five items reflecting care (range 0–3), nursing (range 0–3), therapy disciplines (range 0–3), therapy intensity (range 0–3) and medical (range 0–3) needs. It is designed to be applied either prospectively (to reflect complexity of needs for rehabilitation) or retrospectively (to reflect the level of input provided). In this analysis, RCS data were collected retrospectively to reflect the actual levels of input provided during the preceding week at the time of rating.
- The UK FIM+FAM is a 30-item scale of functional independence (total score range 30–210) comprising 16 motor (range 16–112) and 14 cognitive (range 14–98) items.

### Data handling

Data were extracted from the UKROC database and transferred to SPSS version 19 for analysis. Mean, standard deviation and range scores were calculated for interval data (age, length of stay, costs etc.) and median and interquartile ranges (IQR) for ordinal data. Because a considerable portion of the data were skewed, nonparametric statistical techniques were used throughout for analysis.

### Ethics

This work was undertaken as part of a registered Payment by Results Improvement Project. It involved only the analysis of routinely collected clinical and costing data. As such, it falls under the category of service evaluation for which no research ethics permission is required in the UK. This has been confirmed by peer review through the Research and Development Department at the lead Trust.

## Results

**Figure 1.**
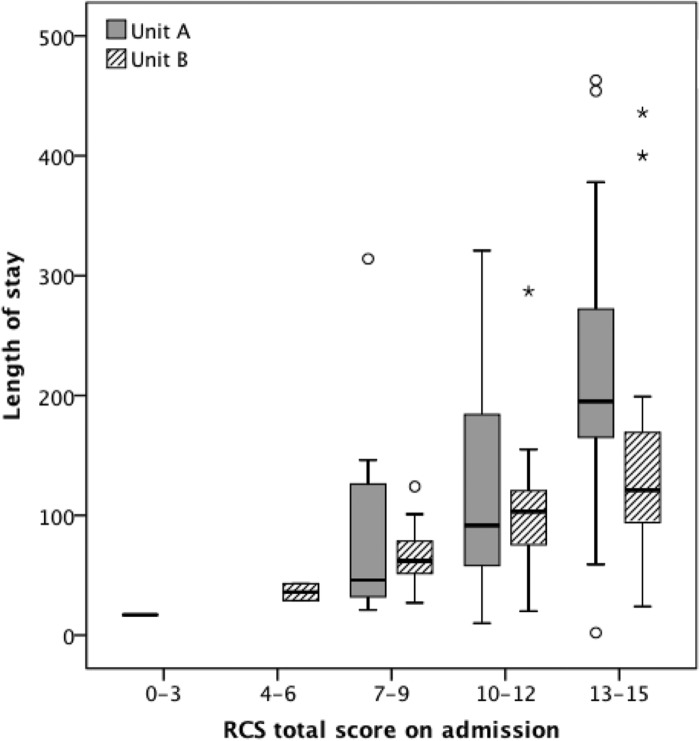
Length of stay compared across different levels of complexity. It shows the breakdown of length of stay for the two units, across five different levels of complexity, based on the total Rehabilitation Complexity Score (RCS) score on admission. The length of stay is consistently higher for Unit A than Unit B at each level.

A total of 173 admissions were recorded: Unit A (n = 82), Unit B (n = 91). Demographic data for the two cohorts are shown in Table I. The mean age for both units was under 50 years, and the diagnostic profile was broadly similar for the two services. Unit A admitted a smaller proportion of stroke patients, due to the presence of a dedicated stroke rehabilita­tion ward within the same hospital. The mean length of stay was one-and-a-half times longer for Unit A than Unit B (Mann–Whitney z = 2.8, p = −0.005). This longer length of stay was seen consistently across all diagnostic groups and all levels of complexity (see Figure 1), and so could not be explained on the basis of differences in casemix between the two units.

**Table I.** Demographics for the two services.

	Unit A (n = 82)	Unit B (n = 91)
Gender: male (%)	56%	59%
Age (years)		
Mean (SD)	48 (13)	42 (14)
Range	16–69	17–68
Length of stay (days)		
Mean (SD)	147 (106)	98 (66.5)
Range	2–463	3–436
Diagnoses n (%)		
Stroke	19 (23.2%)	42 (45.2%)
Other brain injury	34 (41.5%)	34 (36.6%)
Spinal cord injury	2 (2.4%)	5 (5.4%)
Other neurological	17 (20.7%)	12 (12.9%)
Non neurological	10 (12.2%)	–
Episode cost to purchasers		
Mean (SD)	£48,101 (£35,231)	£44,316 (£31,416)
Range	£706–£163,439	£9,437–£205,731

**Figure 2.**
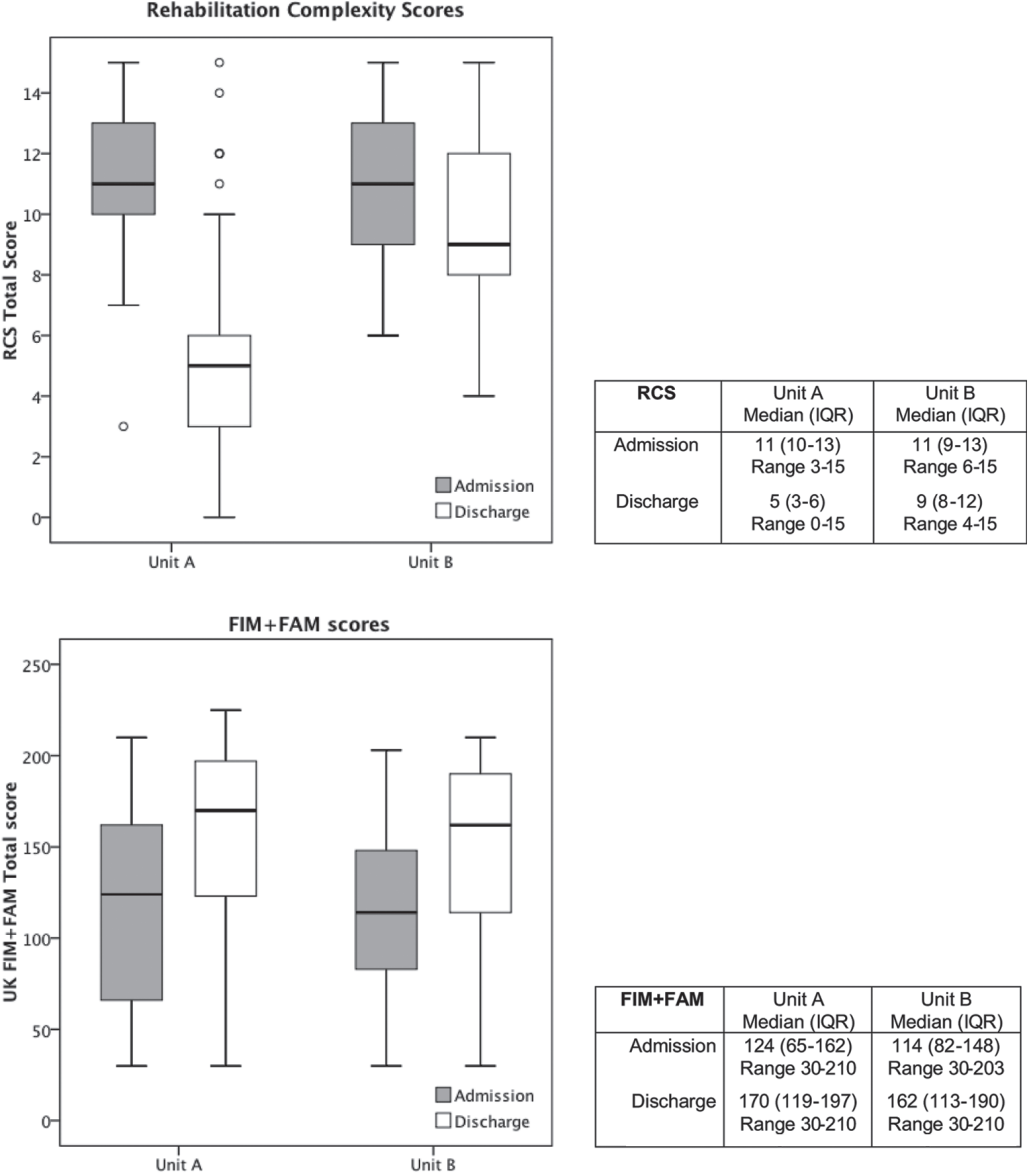
Comparison of total FIM+FAM and RCS scores between the two units. It shows “Box and Whiskersâ plots of total RCS and UK FIM+FAM scores for the two units at admission and discharge.

Total FIM+FAM and RCS (subscale and total) scores were available for all episodes in both units. FIM+FAM motor and cognitive subscale scores were available for all Unit B episodes, but only 10 of the Unit A episodes, so statistical comparison could not be performed at subscale level. Median and IQR for the total scores on admission and discharge are shown in Figure 2.

A summary of statistical analysis within and between the units is shown in Table II. On admission, total RCS and FIM+FAM scores were broadly similar for the two services (see Figure 2). Both scores changed significantly between admission and discharge. On discharge, there was no difference between the units in total FIM+FAM score, but the RCS total score (indicating levels of input) was significantly lower for Unit A (z = −7.9, p < 0.0001). Table III shows a breakdown of the RCS subscale scores and a summary of between group differences at admission and discharge. At discharge, all RCS subscales except Care were significantly lower for Unit A – the most important difference being within the therapy subscales, with lesser differences in the nursing and medical subscales. The most striking difference was that therapy inputs fell between admission and discharge for Unit A whilst they rose in Unit B (see discussion).

**Table II.** Summary of within- and between-unit statistical analysis.

Parameter	Within unit		Between unit	
	Admission to discharge (Wilcoxon Sign rank tests)		(Mann–Whitney tests)	
	Unit A	Unit B	Admission	Discharge
RCS	z = −7.5	z = −6.1	z = −1.2	z = −7.9
Total score	p < 0.001	p < 0.001	p = 0.217	p < 0.001
UK FIM+FAM	z = −7.4	z = −7.3	z = −1.0	z = −1.2
Total score	p < 0.001	p < 0.001	p = 0.298	p = 0.219

RCS, Rehabilitation Complexity Score; UK FIM+FAM, Functional Assessment Measure (UK version).

**Table III.** Breakdown of Rehabilitation Complexity Score (RCS) subscale scores.

RCS subscale	Admission					Discharge		
	Unit A	Unit B	Mann–Whitney		Unit A	Unit B	Mann–Whitney	
	Median (IQR)	Median (IQR)	z	p	Median (IQR)	Median (IQR)	z	p
Care	2 (1–3)	2 (1–2)	−1.9	0.058	1 (0–2)	1 (0–2)	−0.2	0.847
Nursing	2 (2–3)	2 (2–3)	−0.3	0.744	1 (0–1)	2 (1–3)	−6.2	**<0.001**
Therapy disciplines	3 (3–3)	2 (2–3)	−5.1	**<0.001**	2 (1–2)	3 (3–3)	−9.6	**<0.001**
Therapy intensity	3 (2–3)	2 (2–3)	−1.1	0.276	1 (1–1)	3 (2–3)	−9.8	**<0.001**
Medical	2 (2–2)	2 (2–2)	−2.9	**0.004**	0 (0–1)	1 (1–2)	−6.4	**<0.001**
Total	11 (10–13)	11 (9–13)	−1.2	0.217	5 (3–6)	9 (8–12)	−7.9	**<0.001**

A comparison of annual service costs for the two units is given in Table IV. The total costs for each service were remarkably similar, as was the breakdown of cost types (direct, central and overheads). However, there were 30 beds in Unit A, compared with 22 in Unit B, giving a mean occupied bed-day (OBD) cost of £406 for Unit A and £507 for Unit B. When translated into whole episode costs, however, the lower OBD cost for Unit A was cancelled out by the longer length of stay, so that the mean cost per case of providing the service was actually higher for Unit A (£59.7k) than for Unit B (£49.7k). The 2008/9 commissioned prices for both units (i.e. cost to the purchaser) were lower than the actual costs of provision by about 20% for Unit A and 12% for Unit B.

**Table IV.** Comparison of 2008/9 annual service costs for the two units.

	Unit A		Unit B	
Unit running costs in 2008/9				
Direct clinical costs	£3,499k	87%	£3,343k	83%
Central costs and overheads	£505k	13%	£701k	17%
Total unit costs	£4,004	100%	£4,045	100%
No. of beds (mean occupancy)	30 (27)	90%	22 (21.7)	97%
Activity (OBD)	9855		7921	
Mean OBD cost	£406		£507	
Mean length of stay	147 days		98 days	
Mean cost per case to provider	£59,682		£49,686	
Mean cost per case to purchaser (at contract price)	£48,101		£44,316	

OBD, occupied bed-day.

The total staffing levels for the units were almost identical at around 60 whole time equivalents (WTE). A breakdown of staffing is given in Table V. When converted to WTE per occupied bed-day, the two units had approximately equivalent numbers of nursing, medical and admin/managerial staff. However, the therapy staffing levels were strikingly different – approximately 85% higher for Unit B. With the exception of physiotherapy staff, these differences are apparent across all disciplines.

**Table V.** Staffing levels for the two units.

	Unit A		Unit B	
	Occupied bed days = 27		Occupied bed days = 21.7	
	WTE	WTE per OBD	WTE	WTE per OBD
Qualified nurses	15.35	0.57	21.15	0.97
Care assistants	23	0.85	11.6	0.53
Nursing and care staff	**39.25**	**1.45**	**32.75**	**1.51**
Consultants in RM	2	0.07	1.6	0.07
Junior medical staff	2	0.07	2	0.09
Total medical staff	**4**	**0.15**	**3.6**	**0.17**
Admin and clerical	3	0.11	3	0.14
Managerial etc	2	0.07	1.6	0.07
Total	**5**	**0.19**	**4.6**	**0.21**
Therapy staff				
Physiotherapy	7	0.26	6	0.28
Occupational therapy	4	0.15	6	0.28
Speech and language	1.9	0.07	3.6	0.17
Psychology/counselling	1.6	0.06	2.6	0.12
Dietetics	0.2	0.01	1	0.05
Social work	0	0.00	2	0.09
Technical assistants	1	0.04	2	0.09
Total therapy staff	**15.7**	**0.58**	**23.2**	**1.07**
Total staffing	**59**	**2.19**	**60.5**	**2.78**

OBD, occupied bed-day; RM, rehabilitation medicine; WTE, whole time equivalent.

**Figure 3.**
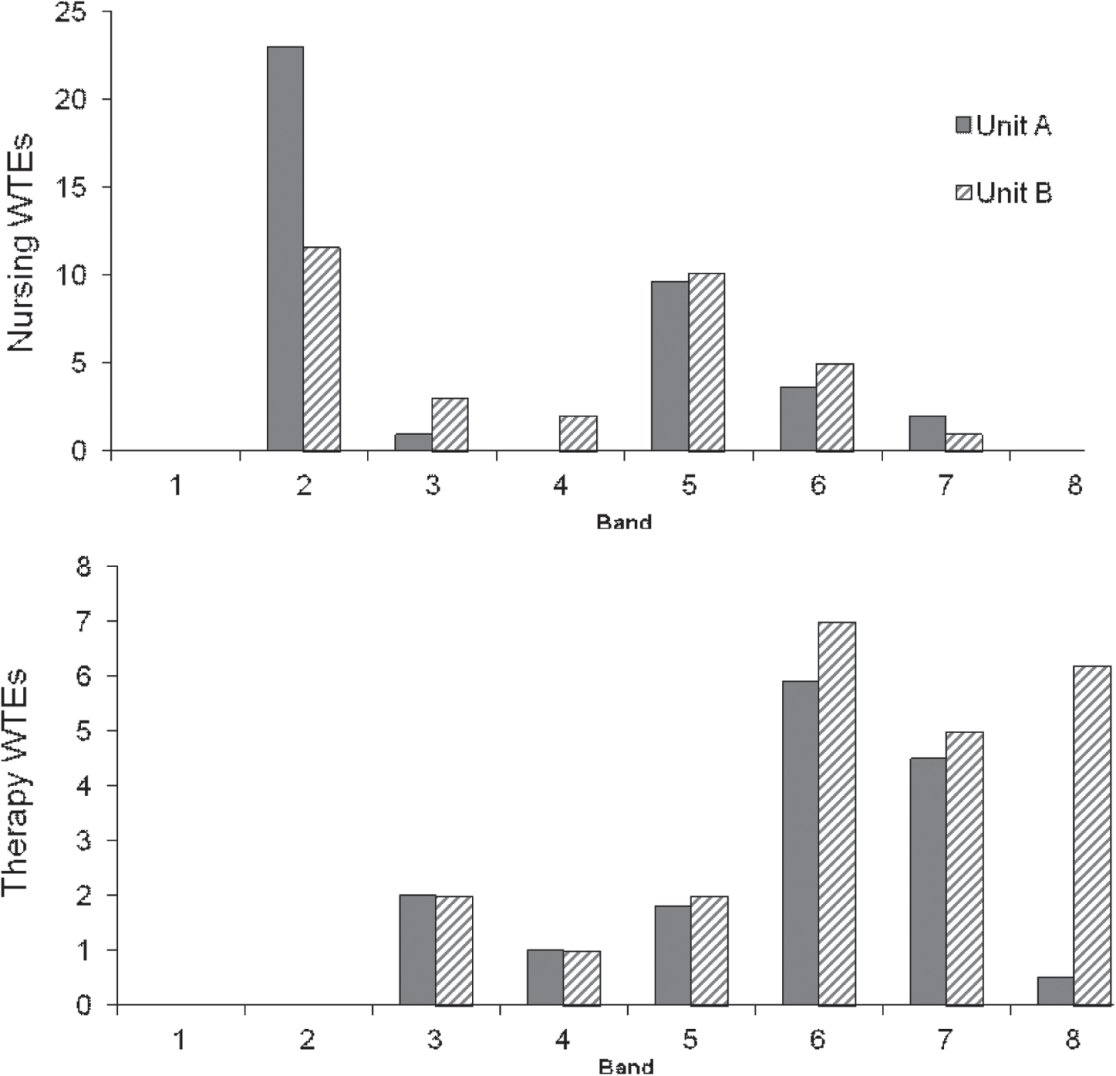
Breakdown of nursing and therapy staff whole time equivalents (WTEs) by banding. It shows the comparative banding of nursing and therapy staff in WTEs for the two units. Unit A has a higher proportion of nonqualified care staff, and a lower proportion of senior therapy staff than Unit B.

Figure 3 shows a breakdown of staff numbers by grade. (In the UK staff banding system, bands 1–4 are nonqualified healthcare assistants; band 5 is the entry level for qualified staff – both in nursing and therapy disciplines. Band 7 represents senior specialist clinical staff, and band 8 staff have managerial responsibilities.) The nursing profile for the two services shows that there was a higher proportion of nonqualified care staff in Unit A, but approximately similar numbers of qualified nursing staff. Within the therapy staffing profile, however, there was a marked difference in the proportion of higher grade posts in favour of Unit B.

## Discussion

In this analysis of data from two specialist rehabilitation units, we have shown that the two services had broadly similar populations, in terms of age, casemix and dependency on admission. In addition the two groups reached a similar level of functional independence by discharge. However, Unit A took approximately one-and-a-half times longer to achieve this gain. Unit A had substantially fewer therapy staff than Unit B and so was less expensive to run. However, any savings in service running costs were cancelled out by the longer lengths of stay, so that the total cost per case was actually lower for Unit B making it more cost-efficient overall.

A notable difference between the two services was the level of intervention at the time of discharge. A reduction in needs for care, nursing and medical intervention would be expected over the course of the rehabilitation programme, and this was reflected in the level of input (as measured by the RCS) for both units. However, there was a striking difference in the level of therapy intervention. For Unit A, therapy inputs dropped off towards the end of the programme, whereas for Unit B therapy inputs were not only sustained, but actually increased towards discharge.

This may be explained by the fact that Unit B is an immediate post-acute rehabilitation ward situated within an acute district hospital. Patients are quite frequently admitted straight from intensive care settings, and many are not fit to engage in an intensive therapy programme immediately on arrival. Because of the shorter length of stay in Unit B, staff typically remain actively engaged in delivering therapy interventions and proactive discharge planning – right up to the point of discharge. By contrast, Unit A is a free-standing rehabilitation hospital, geographically separated from the acute services. Patients generally need to be medically stable before transfer to the Unit. It is possible that, because of longer length of stay, the rehabilitation staff input reduces as the complexity of needs for rehabilitation decreases, and becomes limited to discharge planning by the end of the episode.

Aside from the natural pressures of demand for throughput, there are a number of possible reasons for the longer lengths of stay in Unit A.

- Intensity of therapy intervention is known to impact on the rate of recovery, and a number of studies in the literature have demonstrated reduction in length of stay with more intensive programmes [12–14].
- Unit A did not have a dedicated social worker to assist with discharge planning whereas Unit B had two social workers. Debriefing with staff from the two services revealed that delayed discharges were much more common in Unit A, as staff frequently lacked the time to engage with local housing and social services departments to effect a timely discharge.
- Unit B employed a higher proportion of senior therapy staff in bands 7 and 8. This could simply reflect the need for higher salaries to recruit and retain staff in the London area, where Unit B is situated. However, it may also be expected that these more experienced staff would have the confidence to negotiate with ongoing services and to press for earlier discharge, which may assist timely and efficient discharge planning.
- Unit B operates within a coordinated regional network of services, and there may also be differences in the provision of ongoing rehabilitation and support services in the community that would facilitate earlier discharge for patients in this unit.

The authors recognise a number of limitations to this study.

- Data were gathered during the early part of evolution of the UKROC database, and were incomplete to the extent that only total FIM+FAM scores were available from Unit A, as opposed to item level data, or even motor and cognitive subscales. There may be more subtle differences in the nature of impairments that would impact on length of stay.
- Further analysis is also required to tease out the more subtle differences between the two services, including details arrangements for ongoing rehabilitation/support which are not currently collected as part of the UKROC dataset. These are the subject of a current London-wide study involving Unit B [15].
- Costing data were derived from retrospective analysis of budgets and service accounts rather than true patient-level costing, as few NHS providers in the UK have costing systems sophisticated enough to allocate the direct costs of treatment to individual patients prospectively. Whilst relatively crude, this pragmatic approach [8] provides much more detailed information than is available through the standard UK system of provider-reported “reference costs.â Despite our efforts to standardise the collection of costing data, there may be some differences in the counting of service costs of their attribution to the different cost types.
- Aside from any inaccuracies, retrospective costing systems may also have the disadvantage of being out-of-date even by the time the data are analysed. For example, as noted in Table IV, mean commissioning prices were substantially lower than running costs in 2008/9, especially for the most complex patients. However, the introduction of a weighted bed-day commissioning tariff in 2009/10 for Unit B has since provided a fairer reimbursement system to meet the additional costs of the more complex patients.

These limitations aside, this analysis has highlighted the important role that systematically collected clinical data can play in helping us to understand the factors that underlie cost-efficient service provision or people with complex disability. Importantly, it demonstrates that lower unit front-end costs do not always signify cost-efficiency and that investment in highly skilled therapy staff to deliver an intensive multidisciplinary programme may have the potential to pay for itself through reduced length of stay and increased throughput. Further research is now warranted to find the optimal balance between intensity of input and length of stay for cost-efficient programmes of care and rehabilitation across a wider range of services.

In the meantime, the findings of this analysis were presented to the service managers and commissioners of Unit A. This has led to the development of a four-phase business plan for service reconfiguration. Immediate changes have included the provision of a full-time discharge coordinator and additional investment of approximately £500k to increase the establishment of therapy and nursing staff in from 2011/12. Further plans include the development of community services to support patients after discharge. A future re-evaluation will be conducted when these provisions are in place.

## References

[1] Turner-Stokes L, Williams H, Sephton K Engaging the hearts and minds of clinicians in outcome measurement – the UK Rehabilitation Outcomes Collaborative approach. Disabil Rehabil.

[2] Turner-Stokes L (2008). Evidence for the effectiveness of multi-disciplinary rehabilitation following acquired brain injury: a synthesis of two systematic approaches. J Rehabil Med.

[3] Hoffman JM, Doctor JN, Chan L, Whyte J, Jha A, Dikmen S (2003). Potential impact of the new medicare prospective payment system on reimbursement for traumatic brain injury inpatient rehabilitation. Arch Phys Med Rehabil.

[4] Evans RL, Halar EM, Hendricks RD, Lawrence KV, Kirk C, Bishop DS (1990). Effects of prospective payment financing on rehabilitation outcome. Int J Rehabil Res.

[5] Turner-Stokes L, Paul S, Williams H (2006). Efficiency of specialist rehabilitation in reducing dependency and costs of continuing care for adults with complex acquired brain injuries. J Neurol Neurosurg Psychiatr.

[6] Turner-Stokes L (2007). Cost-efficiency of longer-stay rehabilitation programmes: can they provide value for money?. Brain Inj.

[7] (2002). Reforming NHS financial flows introducing Payment by Results.

[8] Turner-Stokes L, Sutch S, Dredge R (2012). Healthcare tariffs for specialist inpatient neurorehabilitation services: rationale and development of a UK casemix and costing methodology. Clin Rehabil.

[9] Turner-Stokes L, Nyein K, Turner-Stokes T, Gatehouse C (1999). The UK FIM+FAM: development and evaluation. Functional Assessment Measure. Clin Rehabil.

[10] (2009). National Definition Set for Specialised Services No 7: “Complex specialised rehabilitation for brain injury and complex disability (Adult)â.

[11] Turner-Stokes L, Williams H, Siegert RJ (2010). The Rehabilitation Complexity Scale version 2: a clinimetric evaluation in patients with severe complex neurodisability. J Neurol Neurosurg Psychiatr.

[12] Kwakkel G, Wagenaar RC, Koelman TW, Lankhorst GJ, Koetsier JC (1997). Effects of intensity of rehabilitation after stroke. A research synthesis. Stroke.

[13] Kwakkel G (2006). Impact of intensity of practice after stroke: issues for consideration. Disabil Rehabil.

[14] Slade A, Chamberlain MA, Tennant A (1998). Enhancing therapy: does it make a difference?.

[15] Siegert RJ, Turner-Stokes L, McCrone PM, Jackson DM, Playford ED, Fleminger S, Bassett P (2008). Evaluation of community rehabilitation service delivery in long-term neurological conditions (Grant No: 0001833).

